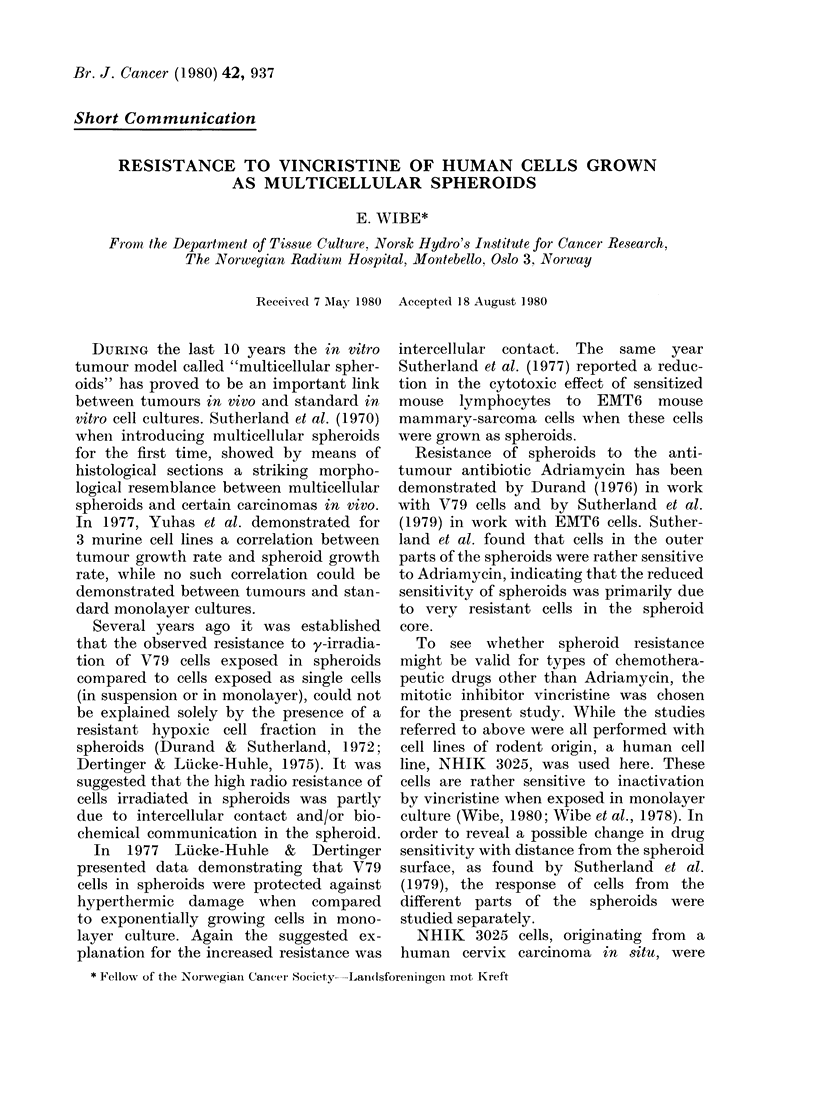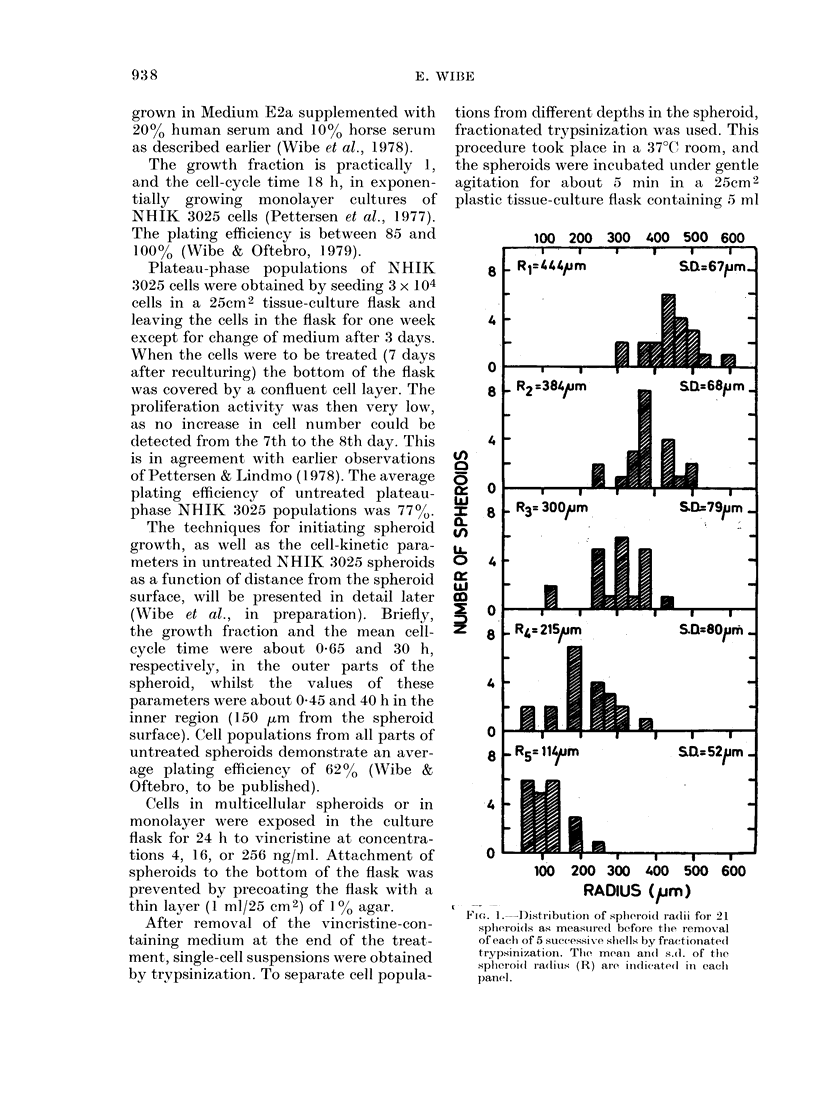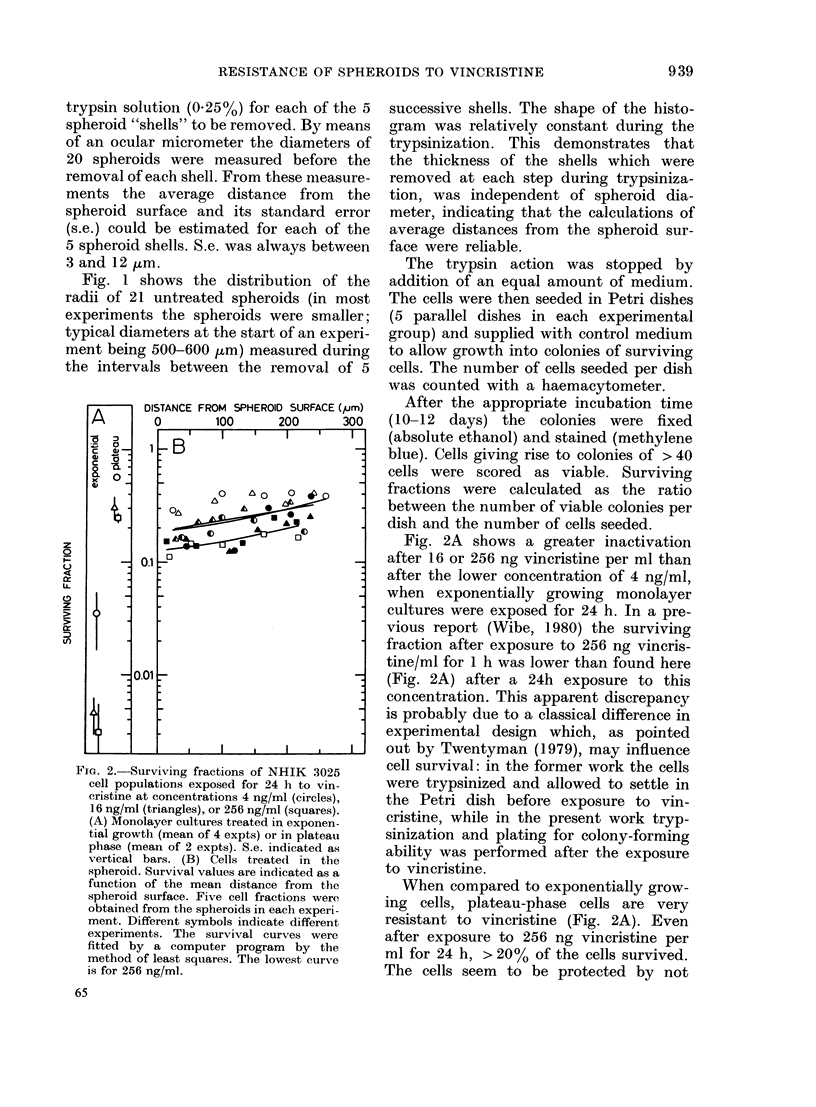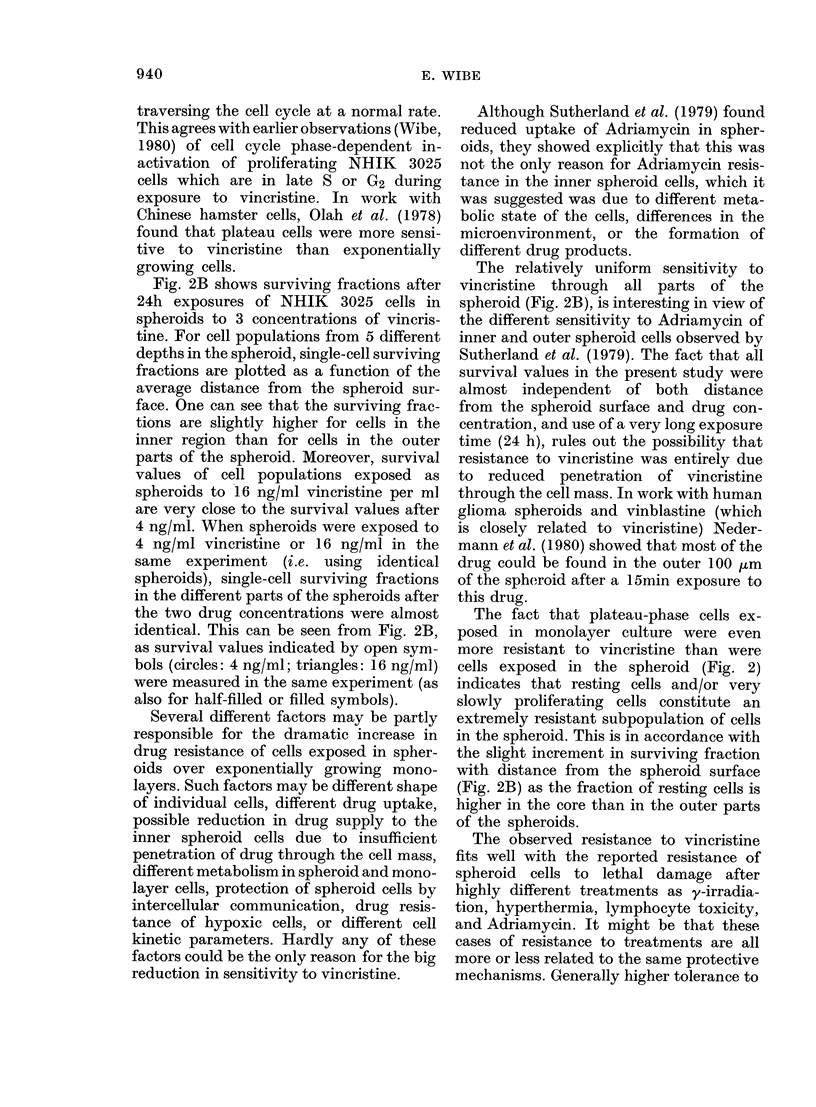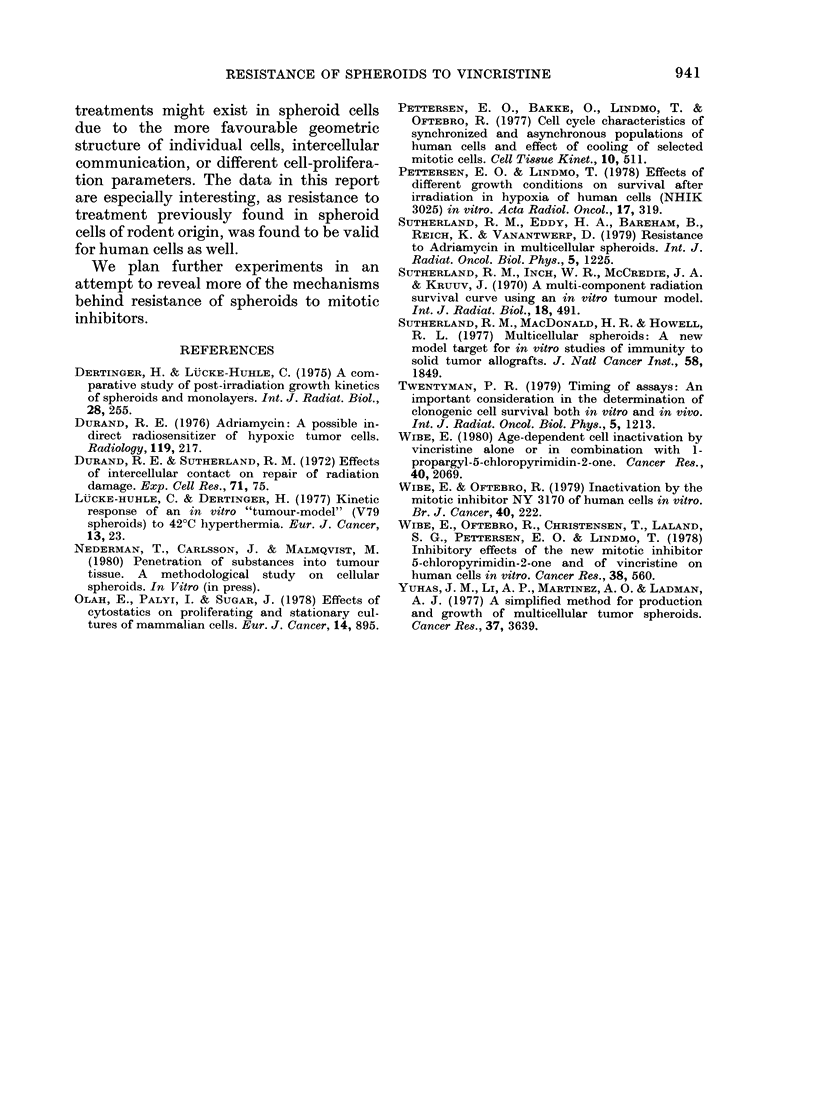# Resistance to vincristine of human cells grown as multicellular spheroids.

**DOI:** 10.1038/bjc.1980.344

**Published:** 1980-12

**Authors:** E. Wibe


*Br. J. Cancer* (1980) **42**, 937

***Short Communication***

### RESISTANCE TO VINCRISTINE OF HUMAN CELLS GROWN AS MULTICELLULAR SPHEROIDS

E. WIBE*

*From the Department of Tissue Culture, Norsk Hydro's Institute for Cancer Research, The Norwegian Radium Hospital, Montebello, Oslo* 3, *Norway*

Received 7 May 1980 Accepted 18 August 1980

DURING the last 10 years the *in vitro* tumour model called "multicellular spheroids" has proved to be an important link between tumours *in vivo* and standard *in vitro* cell cultures. Sutherland *et al.* (1970) when introducing multicellular spheroids for the first time, showed by means of histological sections a striking morphological resemblance between multicellular spheroids and certain carcinomas *in vivo*. In 1977, Yuhas *et al.* demonstrated for 3 murine cell lines a correlation between tumour growth rate and spheroid growth rate, while no such correlation could be demonstrated between tumours and standard monolayer cultures.

Several years ago it was established that the observed resistance to $\gamma$-irradiation of V79 cells exposed in spheroids compared to cells exposed as single cells (in suspension or in monolayer), could not be explained solely by the presence of a resistant hypoxic cell fraction in the spheroids (Durand & Sutherland, 1972; Dertinger & Lücke-Huhle, 1975). It was suggested that the high radio resistance of cells irradiated in spheroids was partly due to intercellular contact and/or biochemical communication in the spheroid.

In 1977 Lücke-Huhle & Dertinger presented data demonstrating that V79 cells in spheroids were protected against hyperthermic damage when compared to exponentially growing cells in monolayer culture. Again the suggested explanation for the increased resistance was intercellular contact. The same year Sutherland *et al.* (1977) reported a reduction in the cytotoxic effect of sensitized mouse lymphocytes to EMT6 mouse mammary-sarcoma cells when these cells were grown as spheroids.

Resistance of spheroids to the antitumour antibiotic Adriamycin has been demonstrated by Durand (1976) in work with V79 cells and by Sutherland *et al.* (1979) in work with EMT6 cells. Sutherland *et al.* found that cells in the outer parts of the spheroids were rather sensitive to Adriamycin, indicating that the reduced sensitivity of spheroids was primarily due to very resistant cells in the spheroid core.

To see whether spheroid resistance might be valid for types of chemotherapeutic drugs other than Adriamycin, the mitotic inhibitor vincristine was chosen for the present study. While the studies referred to above were all performed with cell lines of rodent origin, a human cell line, NHIK 3025, was used here. These cells are rather sensitive to inactivation by vincristine when exposed in monolayer culture (Wibe, 1980; Wibe *et al.*, 1978). In order to reveal a possible change in drug sensitivity with distance from the spheroid surface, as found by Sutherland *et al.* (1979), the response of cells from the different parts of the spheroids were studied separately.

NHIK 3025 cells, originating from a human cervix carcinoma *in situ*, were

* Fellow of the Norwegian Cancer Society—Landsforeningen mot Kreft grown in Medium E2a supplemented with 20% human serum and 10% horse serum as described earlier (Wibe *et al.*, 1978).

The growth fraction is practically 1, and the cell-cycle time 18 h, in exponentially growing monolayer cultures of NHIK 3025 cells (Pettersen *et al.*, 1977). The plating efficiency is between 85 and 100% (Wibe & Oftebro, 1979).

Plateau-phase populations of NHIK 3025 cells were obtained by seeding $3 \times 10^4$ cells in a 25cm$^2$ tissue-culture flask and leaving the cells in the flask for one week except for change of medium after 3 days. When the cells were to be treated (7 days after reculturing) the bottom of the flask was covered by a confluent cell layer. The proliferation activity was then very low, as no increase in cell number could be detected from the 7th to the 8th day. This is in agreement with earlier observations of Pettersen & Lindmo (1978). The average plating efficiency of untreated plateau-phase NHIK 3025 populations was 77%.

The techniques for initiating spheroid growth, as well as the cell-kinetic parameters in untreated NHIK 3025 spheroids as a function of distance from the spheroid surface, will be presented in detail later (Wibe *et al.*, in preparation). Briefly, the growth fraction and the mean cell-cycle time were about 0·65 and 30 h, respectively, in the outer parts of the spheroid, whilst the values of these parameters were about 0·45 and 40 h in the inner region (150 $\mu$m from the spheroid surface). Cell populations from all parts of untreated spheroids demonstrate an average plating efficiency of 62% (Wibe & Oftebro, to be published).

Cells in multicellular spheroids or in monolayer were exposed in the culture flask for 24 h to vincristine at concentrations 4, 16, or 256 ng/ml. Attachment of spheroids to the bottom of the flask was prevented by precoating the flask with a thin layer (1 ml/25 cm$^2$) of 1% agar.

After removal of the vincristine-containing medium at the end of the treatment, single-cell suspensions were obtained by trypsinization. To separate cell populations from different depths in the spheroid, fractionated trypsinization was used. This procedure took place in a 37°C room, and the spheroids were incubated under gentle agitation for about 5 min in a 25cm$^2$ plastic tissue-culture flask containing 5 ml

FIG. 1.—Distribution of spheroid radii for 21 spheroids as measured before the removal of each of 5 successive shells by fractionated trypsinization. The mean and s.d. of the spheroid radius (R) are indicated in each panel.

trypsin solution (0·25%) for each of the 5 spheroid "shells" to be removed. By means of an ocular micrometer the diameters of 20 spheroids were measured before the removal of each shell. From these measurements the average distance from the spheroid surface and its standard error (s.e.) could be estimated for each of the 5 spheroid shells. S.e. was always between 3 and 12 $\mu$m.

Fig. 1 shows the distribution of the radii of 21 untreated spheroids (in most experiments the spheroids were smaller; typical diameters at the start of an experiment being 500–600 $\mu$m) measured during the intervals between the removal of 5 successive shells. The shape of the histogram was relatively constant during the trypsinization. This demonstrates that the thickness of the shells which were removed at each step during trypsinization, was independent of spheroid diameter, indicating that the calculations of average distances from the spheroid surface were reliable.

Fig. 2.—Surviving fractions of NHIK 3025 cell populations exposed for 24 h to vincristine at concentrations 4 ng/ml (circles), 16 ng/ml (triangles), or 256 ng/ml (squares). (A) Monolayer cultures treated in exponential growth (mean of 4 expts) or in plateau phase (mean of 2 expts). S.e. indicated as vertical bars. (B) Cells treated in the spheroid. Survival values are indicated as a function of the mean distance from the spheroid surface. Five cell fractions were obtained from the spheroids in each experiment. Different symbols indicate different experiments. The survival curves were fitted by a computer program by the method of least squares. The lowest curve is for 256 ng/ml.

The trypsin action was stopped by addition of an equal amount of medium. The cells were then seeded in Petri dishes (5 parallel dishes in each experimental group) and supplied with control medium to allow growth into colonies of surviving cells. The number of cells seeded per dish was counted with a haemacytometer.

After the appropriate incubation time (10–12 days) the colonies were fixed (absolute ethanol) and stained (methylene blue). Cells giving rise to colonies of $>40$ cells were scored as viable. Surviving fractions were calculated as the ratio between the number of viable colonies per dish and the number of cells seeded.

Fig. 2A shows a greater inactivation after 16 or 256 ng vincristine per ml than after the lower concentration of 4 ng/ml, when exponentially growing monolayer cultures were exposed for 24 h. In a previous report (Wibe, 1980) the surviving fraction after exposure to 256 ng vincristine/ml for 1 h was lower than found here (Fig. 2A) after a 24h exposure to this concentration. This apparent discrepancy is probably due to a classical difference in experimental design which, as pointed out by Twentyman (1979), may influence cell survival: in the former work the cells were trypsinized and allowed to settle in the Petri dish before exposure to vincristine, while in the present work trypsinization and plating for colony-forming ability was performed after the exposure to vincristine.

When compared to exponentially growing cells, plateau-phase cells are very resistant to vincristine (Fig. 2A). Even after exposure to 256 ng vincristine per ml for 24 h, $>20\%$ of the cells survived. The cells seem to be protected by not traversing the cell cycle at a normal rate. This agrees with earlier observations (Wibe, 1980) of cell cycle phase-dependent inactivation of proliferating NHIK 3025 cells which are in late S or $G_2$ during exposure to vincristine. In work with Chinese hamster cells, Olah *et al.* (1978) found that plateau cells were more sensitive to vincristine than exponentially growing cells.

Fig. 2B shows surviving fractions after 24h exposures of NHIK 3025 cells in spheroids to 3 concentrations of vincristine. For cell populations from 5 different depths in the spheroid, single-cell surviving fractions are plotted as a function of the average distance from the spheroid surface. One can see that the surviving fractions are slightly higher for cells in the inner region than for cells in the outer parts of the spheroid. Moreover, survival values of cell populations exposed as spheroids to 16 ng/ml vincristine per ml are very close to the survival values after 4 ng/ml. When spheroids were exposed to 4 ng/ml vincristine or 16 ng/ml in the same experiment (*i.e.* using identical spheroids), single-cell surviving fractions in the different parts of the spheroids after the two drug concentrations were almost identical. This can be seen from Fig. 2B, as survival values indicated by open symbols (circles: 4 ng/ml; triangles: 16 ng/ml) were measured in the same experiment (as also for half-filled or filled symbols).

Several different factors may be partly responsible for the dramatic increase in drug resistance of cells exposed in spheroids over exponentially growing monolayers. Such factors may be different shape of individual cells, different drug uptake, possible reduction in drug supply to the inner spheroid cells due to insufficient penetration of drug through the cell mass, different metabolism in spheroid and monolayer cells, protection of spheroid cells by intercellular communication, drug resistance of hypoxic cells, or different cell kinetic parameters. Hardly any of these factors could be the only reason for the big reduction in sensitivity to vincristine.

Although Sutherland *et al.* (1979) found reduced uptake of Adriamycin in spheroids, they showed explicitly that this was not the only reason for Adriamycin resistance in the inner spheroid cells, which it was suggested was due to different metabolic state of the cells, differences in the microenvironment, or the formation of different drug products.

The relatively uniform sensitivity to vincristine through all parts of the spheroid (Fig. 2B), is interesting in view of the different sensitivity to Adriamycin of inner and outer spheroid cells observed by Sutherland *et al.* (1979). The fact that all survival values in the present study were almost independent of both distance from the spheroid surface and drug concentration, and use of a very long exposure time (24 h), rules out the possibility that resistance to vincristine was entirely due to reduced penetration of vincristine through the cell mass. In work with human glioma spheroids and vinblastine (which is closely related to vincristine) Nedermann *et al.* (1980) showed that most of the drug could be found in the outer 100 $\mu$m of the spheroid after a 15min exposure to this drug.

The fact that plateau-phase cells exposed in monolayer culture were even more resistant to vincristine than were cells exposed in the spheroid (Fig. 2) indicates that resting cells and/or very slowly proliferating cells constitute an extremely resistant subpopulation of cells in the spheroid. This is in accordance with the slight increment in surviving fraction with distance from the spheroid surface (Fig. 2B) as the fraction of resting cells is higher in the core than in the outer parts of the spheroids.

The observed resistance to vincristine fits well with the reported resistance of spheroid cells to lethal damage after highly different treatments as $\gamma$-irradiation, hyperthermia, lymphocyte toxicity, and Adriamycin. It might be that these cases of resistance to treatments are all more or less related to the same protective mechanisms. Generally higher tolerance to treatments might exist in spheroid cells due to the more favourable geometric structure of individual cells, intercellular communication, or different cell-proliferation parameters. The data in this report are especially interesting, as resistance to treatment previously found in spheroid cells of rodent origin, was found to be valid for human cells as well.

We plan further experiments in an attempt to reveal more of the mechanisms behind resistance of spheroids to mitotic inhibitors.

### REFERENCES

DERTINGER, H. & LÜCKE-HUHLE, C. (1975) A comparative study of post-irradiation growth kinetics of spheroids and monolayers. *Int. J. Radiat. Biol.*, **28**, 255.

DURAND, R. E. (1976) Adriamycin: A possible indirect radiosensitizer of hypoxic tumor cells. *Radiology*, **119**, 217.

DURAND, R. E. & SUTHERLAND, R. M. (1972) Effects of intercellular contact on repair of radiation damage. *Exp. Cell Res.*, **71**, 75.

LÜCKE-HUHLE, C. & DERTINGER, H. (1977) Kinetic response of an *in vitro* "tumour-model" (V79 spheroids) to 42°C hyperthermia. *Eur. J. Cancer*, **13**, 23.

NEDERMAN, T., CARLSSON, J. & MALMQVIST, M. (1980) Penetration of substances into tumour tissue. A methodological study on cellular spheroids. *In Vitro* (in press).

OLAH, E., PALYI, I. & SUGAR, J. (1978) Effects of cytostatics on proliferating and stationary cultures of mammalian cells. *Eur. J. Cancer*, **14**, 895.

PETTERSEN, E. O., BAKKE, O., LINDMO, T. & OFTEBRO, R. (1977) Cell cycle characteristics of synchronized and asynchronous populations of human cells and effect of cooling of selected mitotic cells. *Cell Tissue Kinet.*, **10**, 511.

PETTERSEN, E. O. & LINDMO, T. (1978) Effects of different growth conditions on survival after irradiation in hypoxia of human cells (NHIK 3025) *in vitro*. *Acta Radiol. Oncol.*, **17**, 319.

SUTHERLAND, R. M., EDDY, H. A., BAREHAM, B., REICH, K. & VANANTWERP, D. (1979) Resistance to Adriamycin in multicellular spheroids. *Int. J. Radiat. Oncol. Biol. Phys.*, **5**, 1225.

SUTHERLAND, R. M., INCH, W. R., MCCREDIE, J. A. & KRUUV, J. (1970) A multi-component radiation survival curve using an *in vitro* tumour model. *Int. J. Radiat. Biol.*, **18**, 491.

SUTHERLAND, R. M., MACDONALD, H. R. & HOWELL, R. L. (1977) Multicellular spheroids: A new model target for *in vitro* studies of immunity to solid tumor allografts. *J. Natl Cancer Inst.*, **58**, 1849.

TWENTYMAN, P. R. (1979) Timing of assays: An important consideration in the determination of clonogenic cell survival both *in vitro* and *in vivo*. *Int. J. Radiat. Oncol. Biol. Phys.*, **5**, 1213.

WIBE, E. (1980) Age-dependent cell inactivation by vincristine alone or in combination with 1-propargyl-5-chloropyrimidin-2-one. *Cancer Res.*, **40**, 2069.

WIBE, E. & OFTEBRO, R. (1979) Inactivation by the mitotic inhibitor NY 3170 of human cells *in vitro*. *Br. J. Cancer*, **40**, 222.

WIBE, E., OFTEBRO, R., CHRISTENSEN, T., LALAND, S. G., PETTERSEN, E. O. & LINDMO, T. (1978) Inhibitory effects of the new mitotic inhibitor 5-chloropyrimidin-2-one and of vincristine on human cells *in vitro*. *Cancer Res.*, **38**, 560.

YUHAS, J. M., LI, A. P., MARTINEZ, A. O. & LADMAN, A. J. (1977) A simplified method for production and growth of multicellular tumor spheroids. *Cancer Res.*, **37**, 3639.